# Grazing-Induced Habitat Degradation: Challenges to Giant Panda Survival Resulting from Declining Bamboo and Soil Quality

**DOI:** 10.3390/ani15020202

**Published:** 2025-01-14

**Authors:** Huawei Tian, Ying Zeng, Zejun Zhang, Ming Lu, Wei Wei

**Affiliations:** 1 Key Laboratory of Southwest China Wildlife Resources Conservation (Ministry of Education), China West Normal University, Nanchong 637009, China; tianhwv587@163.com (H.T.);; 2 College of Giant Panda, China West Normal University, Nanchong 637009, China; 3 Liziping Giant Panda’s Ecology and Conservation Observation and Research Station of Sichuan Province, Nanchong 637009, China; 4 CAS Key Laboratory of Animal Ecology and Conservation Biology, Institute of Zoology, Chinese Academy of Sciences, Beijing 100101, China

**Keywords:** giant panda (*Ailuropoda melanoleuca*), grazing, bamboo nutrition, soil physicochemical properties, habitat quality

## Abstract

Grazing is the primary human-induced disturbance affecting giant panda (*Ailuropoda melanoleuca*) habitats and has a severe impact on the long-term sustainability of the giant panda population. To address the lack of quantitative studies on grazing’s impact on habitat quality, we selected China’s most heavily grazed giant panda nature reserve. Utilizing the Maxent model and stoichiometric analysis, we investigated habitat quality degradation caused by grazing and quantified changes in bamboo nutritional quality and soil physicochemical properties. The results indicate that grazing has significantly reduced the suitable habitat area for giant pandas from 101.87 km^2^ to 80.64 km^2^. Specifically, high-suitability habitats declined by 14.14%, moderate-suitability habitats declined by 22.70%, and low-suitability habitats declined by 22.88%. Grazing has forced pandas to move to higher altitudes (2650–3057 m) with taller (12–20 m) trees, denser (28–55 plants) shrubs, and sparser (30–69%) bamboo. Additionally, the soil water content has decreased, while soil bulk density, total N, available N, and pH have significantly increased. Reductions in crude protein and ether extract, along with increased crude fiber and ash, have lowered bamboo’s nutritional value and palatability. This study elucidates how grazing degrades giant panda habitat quality and provides a scientific basis for its conservation management.

## Simple Summary

Giant pandas are endangered animals that rely on bamboo forests for their survival. However, livestock grazing has become the main threat to these forests, putting the pandas’ long-term future at risk. This study was conducted in China’s most heavily grazed nature reserve to determine how grazing affects pandas’ habitats, especially the bamboo they forage and the soil in which it grows. We used scientific methods to explain changes in the environment caused by grazing. We found that grazing has significantly reduced the area of suitable habitats for pandas, forcing them to move to higher and less ideal areas with taller trees, more shrubs, and less and shorter bamboo. In addition, grazing has caused the soil to become dry and hard, which hinders bamboo growth and makes it less nutritious and palatable for pandas. Our findings help illustrate how grazing damages panda habitats and provide valuable information for conservation efforts. By managing grazing activities and restoring bamboo forests and vegetation, we can better protect panda populations and ensure their habitats remain healthy and sustainable.

## 1. Introduction

Since the 20th century, escalating human activities have severely impacted the survival of wildlife, threatening global biodiversity [1]. Currently, grazing by livestock such as cattle and sheep is one of the most significant human-induced disturbances, profoundly affecting forest ecosystems [2] and the long-term survival of many wildlife populations [3]. Through behaviors such as browsing (livestock feeding on plants), trampling (livestock stepping on or crushing vegetation and soil), and brushing (livestock using their bodies to push against or rub on vegetation), grazing disrupts plant community structures and ecological functions [4] and alters the soil environment [5], thus having a significant impact on forest ecosystems [2,6]. Ellison found that grazing livestock selectively forage for plants, leading to significant reductions in the biomass and abundance of more palatable plants, ultimately resulting in plant communities dominated by less palatable species [7]. Moreover, extensive livestock activities influence nutrient cycling within habitats [8], increase interspecies competition for resources [9], cause land degradation and water and soil pollution [10], and contribute to decreased biodiversity [11]. In an Andean semi-desert in South America, Borgnia’s study indicated that in habitats co-occupied by livestock and vicuñas (*Vicugna vicugna*), livestock occupied the optimal areas, forcing vicuñas to settle for suboptimal habitats [3].

The giant panda (*Ailuropoda melanoleuca*), as a rare endemic relict species unique to China, is also a flagship species for global biodiversity conservation [12]. Through a long evolutionary process, it has specialized in feeding on bamboo [13], which has narrowed its ecological niche and limited its survival range due to food source constraints. The current wild population numbers about two thousand individuals, distributed only in China’s six major mountain ranges: Qinling, Minshan, Qionglai, Daxiangling, Xiaoxiangling, and Liangshan [14,15]. Ultimately, thirty-three highly fragmented local populations have formed. Factors affecting the survival of giant panda populations and habitat degradation include natural phenomena and disasters (periodic flowering of bamboo, geological disasters such as earthquakes and mudslides, forest fires, droughts, snow disasters, and other climate change-related events) [16]. In addition, the most significant stress results from disturbances related to human activity. Livestock grazing has replaced logging as the most severe human disturbance factor within giant panda habitats. It spans a large elevation range (1500–3600 m), has a wide distribution (all giant panda distribution areas), and has a high encounter rate in the wild (up to 40%) [14].

Grazing impacts giant pandas mainly by way of livestock invading their habitats, compressing their ecological niches, competing for food resources, trampling vegetation, and ultimately leading to a decline in habitat quality [17,18,19]. Previous studies have shown that giant pandas significantly avoid areas with high grazing intensity, preferring areas with low grazing intensity or no grazing disturbance, which has led to the shifting of giant panda populations towards higher-elevation areas [20]. Giant pandas prefer to select bamboo forests within their habitats that are growing well and have high coverage, completely avoiding habitats with little or no bamboo [21]. Grazing not only significantly reduces bamboo forest coverage but also indirectly affects the height, size, and quantity of bamboo by altering the soil microenvironment [22], ultimately impacting the regeneration and growth of bamboo [23,24,25]. Bamboo is the primary food source for giant pandas and plays a vital role in their survival, with pandas spending 67% of the year primarily consuming bamboo leaves and culms [13,14,25]. Numerous studies have focused on the nutrients in the staple bamboo of giant pandas, measuring the nutritional quality—such as crude protein, ether extract, and crude fiber—of bamboo from different mountain ranges [12,26,27,28]. Giant pandas typically select bamboo leaves and stems that are high in protein and low in fiber content to meet their daily nutritional needs [29,30]. Grazing reduces the crude protein and ether extract while increasing the contents of crude fiber and other nutritional components in bamboo [17], significantly affecting the quality of the giant pandas’ food resources. To resist herbivore predation, plants usually secrete secondary metabolites [31]; the accumulation of these secondary metabolites not only affects the foraging strategies of animals [32] but also impacts their growth and development [33].

Currently, research on grazing livestock and giant pandas mainly focuses on the overlap or differentiation of their ecological niches, comparisons of behavioral patterns and activity patterns between the two, the impact on the abundance and distribution of sympatric species of giant pandas, and the degree of encroachment on giant panda habitats and the extent of bamboo forest destruction. However, there is a lack of systematic research exploring the impact of grazing on the quality of giant panda habitats, especially regarding the quantitative analysis of bamboo nutritional components, secondary metabolites, and soil physicochemical properties. Therefore, in this study, we selected the wild giant panda habitat with the most severe grazing as the research area. Using bamboo nutritional quality, bamboo secondary metabolites, and soil physicochemical properties as important indicators, we evaluated the quality of giant panda habitats. By comparing the habitats of grazing livestock and giant pandas (in their natural state), we analyzed the significant differences in habitat characteristics, bamboo nutritional components, and soil physicochemical properties to explore the specific impacts of grazing on the quality of giant panda habitats. Under grazing disturbance, we examined the specific changes in bamboo nutritional quality and soil physicochemical properties within the habitat. The results can provide a scientific basis for the healthy survival of giant panda populations and the protection and restoration of their habitats.

## 2. Materials and Methods

### 2.1. Study Area

**Figure 1:**
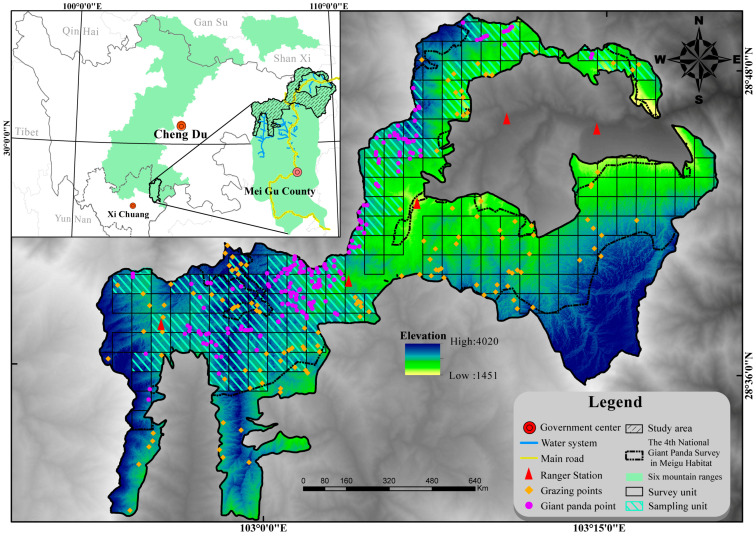
Meigu Dafengding National Nature Reserve, Sichuan, China.

According to the Fourth National Giant Panda Survey [14,15], grazing disturbances in the Liangshan Mountain Range have the highest encounter rate among all giant panda habitats, reaching 55.46%. Meigu Dafengding National Nature Reserve (102°52′–103°20′ E, 28°30′–28°50′ N, Figure 1) is not only one of the reserves with the most severe grazing in the Liangshan Mountain Range but is also the area with the highest giant panda population density in this mountain range (0.0543 individuals/km^2^) [14], making it typically representative. The reserve is located in Meigu County, Liangshan Yi Autonomous Prefecture, Sichuan Province, with a total area of 506.55 km^2^. Situated in the middle section of the Hengduan Mountains on the southeastern edge of the Qinghai–Tibet Plateau, it is a global biodiversity hotspot [34]. Established in 1978, primarily to protect giant pandas and other rare wild animals and plants, the study area currently hosts 28–29 wild giant pandas [35], with a total habitat and potential habitat area of 365.46 km^2^ [14]. Wild giant pandas are mainly distributed in densely forested mountainous areas at altitudes of 2600 to 3500 m, so these regions have isolated transportation, relatively lagging economic development, and limited production and lifestyles. Following China’s promulgation of the Grain for Green Program and the Natural Forest Protection Project [36,37], which strictly control human disturbances such as forest logging and bamboo shoot harvesting, Meigu County has gradually developed animal husbandry as its pillar industry [38]. Regarding agricultural production, animal husbandry accounts for more than 40% of the county’s economy [39]. In 2018 alone, the local beef cattle stock reached 79,200 head; the sheep stock reached 450,000 head, and the annual total demand for forage amounted to 666,000 tons [40].

### 2.2. Field Data Collection

In the study area, we established several 2 km^2^ survey units based on the Meigu giant panda habitat boundaries outlined in the Fourth National Giant Panda Survey [41]. These units were set up taking into account factors such as topography, vegetation distribution, altitude gradients, grazing activities, giant panda behavior, and the distribution of food resources. Between 2022 and 2023, we laid out 25 survey transects within these units, each measuring 5 to 10 km in length and spaced approximately 1 km apart [38]. The transects were designed to cover all habitat types within the reserve as comprehensively as possible. In accordance with the methods described in [42,43], when evidence of giant panda activity (e.g., feeding sites, feces, resting areas, or maternal dens) was detected, 20 m × 20 m giant panda habitat plots (GPHPs) were immediately established. This process resulted in the creation of a total of 440 GPHPs.

When signs of grazing activity (e.g., livestock presence, droppings, or grazing marks) were detected during the survey, grazing disturbance plots (GDPs) of the same size were established, resulting in a total of 898 GDPs. In both types of plots, species activity sites were recorded, along with detailed information on geography, vegetation, and human disturbances. Additionally, five 1 m × 1 m bamboo subplots were set up at the center and four corners of each GPHP and GDP. In these bamboo subplots, detailed data on bamboo species, the number of shoots, and plant height were recorded. Samples of the giant panda’s primary bamboo species and understory soil were also collected, labeled, and brought back to the laboratory for further analysis (details on the bamboo species and sampling methods are provided later.).

### 2.3. Habitat Suitability

To comprehensively analyze the effects of grazing on giant panda habitats, we used the activity points of both giant pandas and livestock. In addition to field surveys, we incorporated activity points identified during patrols conducted in the reserve from 2022 to 2023. Two hypotheses were proposed: Hypothesis 1 (**H1**) assumed that giant panda habitats were free from any human disturbance (natural state), while Hypothesis 2 (**H2**) assumed that grazing was the sole human disturbance within the giant panda habitats. The purpose of these hypotheses was to simulate the impact of grazing on giant panda habitats under natural conditions using the MaxEnt 3.4.4 species distribution model, which is primarily based on sample locations and environmental covariates [44]. The model outputs a species probability distribution map as a habitat suitability index (HSI), ranging from 0 (unsuitable) to 1 (highly suitable). To avoid excessive collinearity among covariates, we used the Pearson correlation coefficient matrix (|*r*| ≥ 0.85) in ENMTools 3.3 to exclude highly correlated covariates. Seven covariates were ultimately selected, comprising climate (annual precipitation), topography (determined based on the digital elevation model (DEM), aspect, and slope), vegetation (normalized difference vegetation index (NDVI)), China land cover dataset (CLCD), and grazing (Table S1). H2 included all seven covariates, while H1 retained six covariates, excluding grazing. All factor layers were resampled to a 30 m resolution. A total of 75% of the giant panda data points were used as training data, while the remaining 25% were used for testing. The model’s accuracy was validated through 10 bootstrap iterations using AUC values, with scores ranging from 0.9 to 1.0 considered excellent [45]. The Maximum Training Sensitivity Plus Specificity Cloglog Threshold was applied to the HSI to classify habitats as suitable or unsuitable [46]. Considering differences in habitat classification methods, we reclassified suitable habitats as high-suitability habitat, moderate-suitability habitat, and low-suitability habitat using Jenks Natural Breaks in ArcGIS v.10.8 (ESRI, West Redlands, CA, USA) and completed the mapping [47].

### 2.4. Habitat Selection

Geographic (elevation, slope, slope position, aspect), vegetation (number of trees, tree height, tree diameter at breast height (DBH), tree canopy closure, number of shrubs, shrub height, shrub cover), and bamboo (bamboo height, bamboo cover, bamboo growth status, number of dead bamboo plants, and number of bamboo shoots) characteristics from the GPHPs and GDPs were vectorized. The differences in habitat selection between giant pandas and livestock were analyzed based on significant inter-group differences.

### 2.5. Bamboo Nutritional Quality and Secondary Metabolite Analysis

The reserve is home to three major bamboo species consumed by giant pandas: *Bashania faberi* (Rendle) T.P.Yi (elevation: 2845–3771 m), *Yushania ailuropodina* T.P.Yi (elevation: 1356–2821 m), and *Yushania brevipaniculata* (Handel.-Mazzetti) T.P.Yi (elevation: 2454–3297 m) [48]. Based on actual survey plots, we selected areas corresponding to or adjacent to giant panda activities and grazing activities as sampling sites to eliminate errors caused by altitude differences. Within each sampling plot, we collected these three bamboo species and classified the leaves and culms according to their age into annual, biennial, and perennial stages, then processed them into powdered samples for analysis [49]. A total of 403 bamboo samples were collected, including 129 leaf samples and 260 culm samples.

By measuring the content of conventional nutritional components in bamboo samples, such as dry matter, crude protein, crude fiber, crude ash, and ether extract [50], as well as secondary metabolites such as tannins, flavonoids, and polyphenols [51,52,53], we compared the significant differences between GPHPs and GDPs to explore the overall impact of grazing on bamboo nutritional quality and secondary metabolites (Table S2). To further evaluate the impact of grazing on the conventional nutritional components of bamboo leaves and culms consumed by giant pandas, we categorized bamboo leaves and culms into six stages based on bamboo age (annual leaf (AL), biennial leaf (BL), perennial leaf (PL), annual culm (AC), biennial culm (BC), and perennial culm (PC)). By measuring the conventional nutritional components of bamboo samples, we analyzed the significant differences between GPHPs and GDPs.

### 2.6. Soil Physicochemical Properties

In each bamboo subplot, natural soil samples were uniformly collected using a 100 cm^3^ ring knife. Sampling was repeated three times, and the soil samples from the same plot were thoroughly mixed, sealed, and numbered so that the soil bulk density, capillary porosity, and capillary moisture capacity could be measured. Fresh soil samples of 10 g–20 g were crumbled and quickly placed into pre-weighed, numbered aluminum boxes to determine the soil water content. Soil samples weighing 200 g were collected to measure the amount of soil available N, available K, pH value, available P, total N, and total C [53] (Table S3). When analyzing soil’s physicochemical properties, we extracted and classified the bamboo subplots with dead bamboo in each GDP as bamboo death plots (BDPs). By conducting multiple comparisons (based on the least significant difference (LSD)) of soil physicochemical properties among GPHPs, GDPs, and BDPs, we explored the significant differences in the soil physicochemical properties of bamboo according to the following three scenarios: bamboo death caused by grazing disturbance, presence of grazing disturbance, and absence of grazing disturbance.

### 2.7. Data Analysis

Statistical analyses were conducted using IBM SPSS Statistics 26 (IBM Corp., Armonk, NY, USA). The normality of all sample data was evaluated using the Kolmogorov–Smirnov test and the Shapiro–Wilk test. For habitat selection, bamboo nutritional quality, and secondary metabolite data, independent sample t-tests were used to analyze significant differences between two groups if the data followed a normal distribution. If the data did not follow a normal distribution, the Mann–Whitney U test was applied. Given the large sample size of the habitat data, Cohen’s d was used to measure the effect size for parametric tests, and Cliff’s delta was used to measure the effect size for non-parametric tests to mitigate the influence of large sample sizes on significance. For soil physicochemical properties data among the three groups (GPHP, GDP, and BDP), normality tests were also conducted first. For normally distributed data with homogeneity of variances, one-way ANOVA was used to test significant differences among multiple groups. For non-normally distributed data, the Kruskal–Wallis H test was used. For indicators with *p* < 0.05, pairwise comparisons were conducted using the Mann–Whitney U test, and *p*-values were adjusted using the Bonferroni correction to control for Type I errors due to multiple comparisons.

Data are presented as means ± standard deviations (SD) for normally distributed data and as medians (Q1, Q3) for non-normally distributed data. Significance levels are denoted by * for *p* < 0.05, ** for *p* < 0.01, and *** for *p* < 0.001. Statistical graphs were generated using Origin 2022 (OriginLab Corp., Northampton, MA, USA).

## 3. Results

### 3.1. Impact on Habitat Suitability and Selection of Giant Pandas

**Figure 2:**
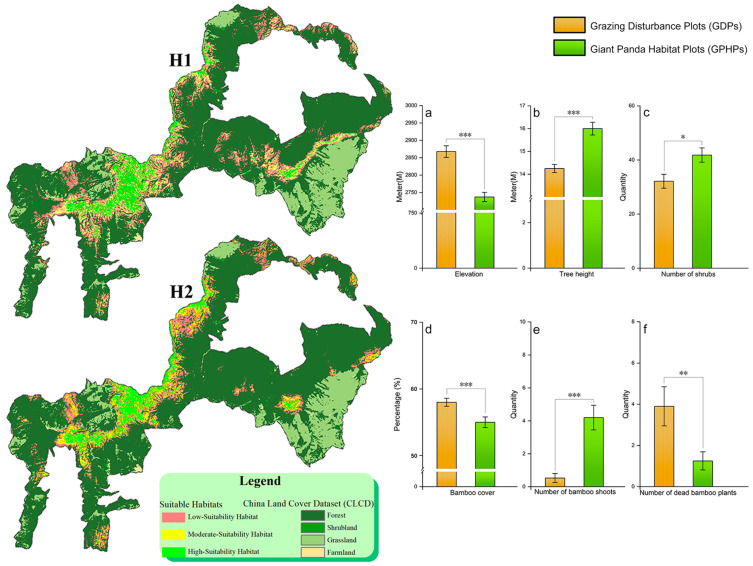
Changes in the suitable habitat of giant pandas and differences in habitat selection under grazing influence. (**H1**) refers to the habitat area of giant pandas in a natural state, with no human disturbance. (**H2**) refers to the habitat area of giant pandas with grazing as the only human disturbance. a-i represent habitat indicators where there are significant differences between livestock and giant pandas, (**a**) is elevation, (**b**) is tree height, (**c**) is the number of shrubs, (**d**) is bamboo coverage, (**e**) is the number of bamboo shoots, and (**f**) is the number of bamboo plants. Due to differences in the normality of data for different habitat indicators, Mean ± Standard Error (SE) is used to draw bar charts to represent the central tendency of the data; error bars indicate ± SE. Significance levels: * *p* < 0.05, ** *p* < 0.01, *** *p* < 0.001, indicating significant differences.

In this study, we employed a scenario hypothesis approach using the MaxEnt model to evaluate the impact of grazing on giant panda habitat suitability within the reserve. The AUC values under both hypotheses exceeded 0.9, indicating excellent model accuracy. Under H1 (natural state), the total suitable habitat area for giant pandas was 101.87 km^2^, comprising 23.19 km^2^ of highly suitable habitat, 30.97 km^2^ of moderately suitable habitat, and 47.71 km^2^ of low-suitability habitat (Figure 2-**H1**). Under H2 (presence of grazing disturbance), the total suitable habitat area significantly decreased to 80.64 km^2^, a reduction of 20.85%. Specifically, the area of highly suitable habitat decreased to 19.91 km^2^ (a 14.14% reduction), moderately suitable habitat shrank to 23.94 km^2^ (a 22.70% reduction), and low-suitability habitat decreased to 36.79 km^2^ (a 22.88% reduction) (Figure 2-**H2**). These results indicate that grazing disturbance leads to a significant reduction in the suitable habitat area for giant pandas, with moderate- and low-suitability habitats being most affected.

The comparative analysis of habitat characteristics further revealed the impact of grazing on the habitat selection of giant pandas. By comparing the habitat characteristics between giant panda habitat plots (GPHPs) and grazing disturbance plots (GDPs), the Cliff’s delta effect size analysis detected significant differences in multiple indicators. Giant pandas preferred areas with higher elevation (N = 1338, *Z* = −5.91, *p* < 0.001;), taller trees (N = 1228, *Z* = −5.21, *p* < 0.001), and a greater number of shrubs (N = 57, *t* = −2.61, *p* < 0.05). In addition, bamboo cover (N = 1303, *Z* = 3.72, *p* < 0.001) and the number of dead bamboo plants (N = 54, *Z* = 2.71, *p* < 0.01) were significantly higher in GDPs than in GPHPs. However, the number of bamboo shoots (N = 55, *Z* = −4.63, *p* < 0.001) was significantly lower in GDPs compared to GPHPs. This indicates that under grazing disturbance, giant pandas are forced to select bamboo forest habitats with lower bamboo cover to avoid areas with a higher number of dead bamboo plants and fewer bamboo shoots (Figure 2a–f, Table S4).

It is also important to note that while tree diameter at breast height (N = 1223, *Z* = −2.90, *p* < 0.01, |δ| = 0.102), bamboo height (N = 1312, *Z* = 2.02, *p* < 0.05, |*δ*| = 0.065), and bamboo growth status (N = 1309, *Z* = −4.80, *p* < 0.001, |*δ*| = 0.048) showed statistical significance between the two plots, the Cliff’s delta effect size values (|*δ*| < 0.11) suggest that these differences may lack substantial ecological significance [54].

### 3.2. Impact on the Nutritional Quality of Bamboo

**Figure 3:**
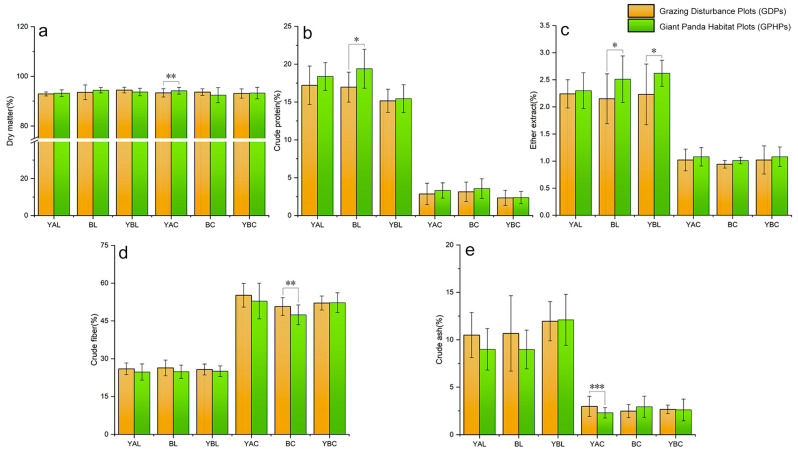
Changes in nutritional quality of leaves and culms of three staple bamboo species for giant pandas under grazing impact (Nutritional quality data were tested for normality, and most data showed normal distribution. Therefore, the Mean ± Standard Deviation (SD) were used to construct bar charts with error bars to illustrate the changes in nutritional quality. Error bars indicate ± SD. Abbreviations in the figure can be explained as follows: YAL: *Yushania ailuropodina* leaves; BL: *Bashania faberi* leaves; YBL: *Yushania brevifolia* leaves; YAC: *Yushania ailuropodina* culms; BC: *Bashania faberi* culms; YBC: *Yushania brevifolia* culms. (**a**). dry matter; (**b**). crude protein; (**c**). ether extract; (**d**). crude fiber; (**e**). crude ash. Significance levels: * *p* < 0.05, ** *p* < 0.01, *** *p* < 0.001, indicating significant differences).

The results of the nutritional quality analysis of leaves and culms from the three bamboo species showed that under grazing conditions, the dry matter content of Y.ailuropodina culms in GDPs was significantly lower than that in GPHPs (N = 109, *t* = −2.38, *p* < 0.01) (Figure 3a), but the crude ash content was significantly higher (N = 76, *t* = 3.15, *p* < 0.001) (Figure 3e). The crude protein (N = 53, *t* = −2.22, *p* < 0.05) and ether extract (N = 33, *t* = −2.28, *p* < 0.05) content of B.faberi leaves in GDPs were significantly lower than those in GPHPs (Figure 3b,c), whereas the crude fiber content of B.faberi culms in GDPs was significantly higher than that in GPHPs (N = 78, *t* = 2.27, *p* < 0.01) (Figure 3d). The ether extract content of Y.brevipaniculata leaves in GDPs was significantly higher than that in GPHPs (N = 41, *Z* = −2.22, *p* < 0.05) (Figure 3c).

The analysis of different age classes of leaves and culms from the three bamboo species showed that grazing had significant effects on the nutritional components of Y.ailuropodina (except for ether extract). In annual culms, the dry matter content in GPHPs was significantly higher than that in GDPs (*t* = −2.26, *p* < 0.05) (Figure S1a). In perennial culms and leaves, the crude protein content in GPHPs was significantly higher than that in GDPs (*Z* = −2.17, *p* < 0.05; *t* = −4.90, *p* < 0.05) (Figure S1b). In biennial culms, the crude fiber content in GPHPs was significantly lower than that in GDPs (*t* = 2.54, *p* < 0.05) (Figure S1d). In perennial leaves, the crude ash content in GPHPs was significantly lower than that in GDPs (*t* = 3.52, *p* < 0.05) (Figure S1e). For B faberi, grazing had significant effects on its nutritional components (except for dry matter and crude ash). In biennial culms, the crude protein content in GPHPs was significantly higher than that in GDPs (*t* = −2.89, *p* < 0.05) (Figure S2b), and the crude fiber content in GPHPs was significantly lower than that in GDPs (*t* = 3.28, *p* < 0.05) (Figure S2d). In perennial culms, the ether extract content in GPHPs was significantly lower than that in GDPs (*t* = −3.10, *p* < 0.05) (Figure S3c). In annual leaves, the crude fiber content in GDPs was higher than that in GPHPs (*t* = 2.54, *p* < 0.05) (Figure S2d). For Y.brevipaniculata, grazing only affected the ether extract content of perennial leaves, with those in GPHPs showing a significantly higher content than those in GDPs (*Z* = 2.94, *p* < 0.01) (Figure S3c).

In summary, our analysis of the five nutritional components of the three primary bamboo species consumed by giant pandas revealed that in response to grazing disturbance, the crude protein and ether extract content of bamboo significantly decreased, but the crude fiber and crude ash content significantly increased. The impact on the nutritional quality was particularly pronounced for Y.ailuropodina.

### 3.3. Impact on Secondary Metabolites

The measurements of secondary metabolites in the leaves and culms of the three bamboo species in GPHPs and GDPs showed that Y.brevipaniculata had higher secondary metabolite content than the other two bamboo species. Regardless of bamboo species, the secondary metabolite contents in bamboo leaves were higher than those in culms. Under grazing conditions, only the flavonoid content showed significant changes. Specifically, in the culms of Y.ailuropodina, the flavonoid content in GDPs was significantly lower than that in GPHPs (N = 18, *t* = −2.37, *p* < 0.05), whereas in the culms of B.faberi, the flavonoid content in GDPs was significantly higher than that in GPHPs (N = 18, *t* = 2.59, *p* < 0.05) (Table 1, Tables S5 and S6, and Figure S4).

### 3.4. Impact on Soil Physicochemical Properties

**Figure 4:**
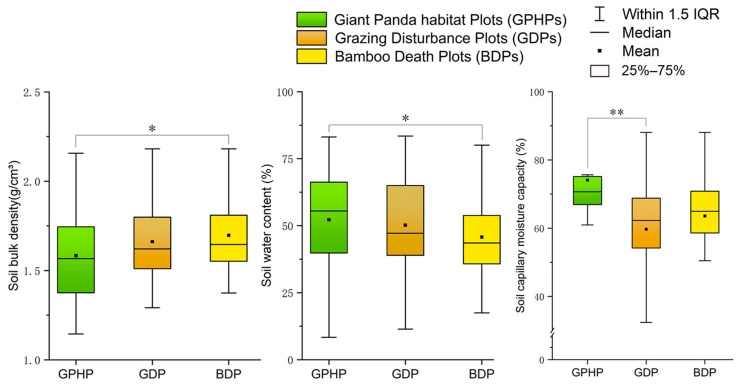
Differences in soil physical properties among three plots under grazing influence. GPHP refers to the giant panda habitat plot, GDP refers to the grazing disturbance plot, and BDP refers to the bamboo death plot. The three plots were compared pairwise using the non-parametric Mann–Whitney U test, and the results are presented using box plots. Significance levels: * *p* < 0.05, ** *p* < 0.01, indicating significant differences.

**Figure 5:**
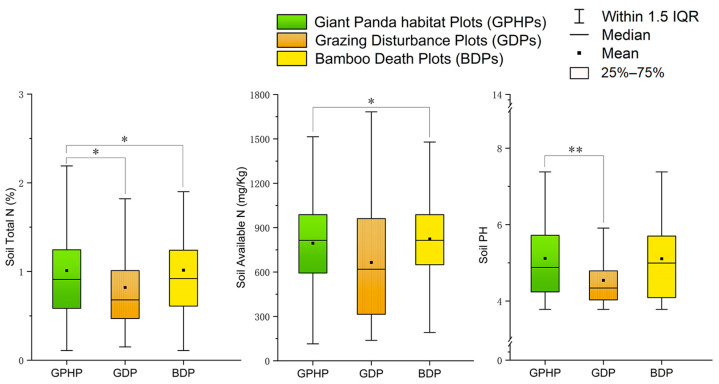
Differences in soil chemical properties among three plots under grazing influence (GPHP refers to the giant panda habitat plot, GDP refers to the grazing disturbance plot, and BDP refers to the bamboo death plot. The three plots were compared pairwise using the non-parametric Mann–Whitney U test, and the results are presented using box plots. Error bars indicate +/− 1.5 IQR. Significance levels: * *p* < 0.05, ** *p* < 0.01, indicating significant differences.

By analyzing 272 soil samples from the three types of plots (GDP, GPHP, and BDP), we found that under grazing conditions, the soil water content in GPHPs was significantly higher than that in BDPs (N = 259, *H* = 6.59, *p* < 0.05). The soil capillary moisture capacity in GPHPs was significantly higher than that in GDPs (N = 126, *H* = 10.53, *p* < 0.01), whereas the soil bulk density in GPHPs was significantly lower than that in BDPs (N = 227, *H* = 6.52, *p* < 0.05) (Figure 4; Table S7). Only soil capillary porosity showed no significant differences among the groups. In terms of soil chemical properties, the contents of soil total C, available K, and available P did not differ significantly among the three types of plots. Under grazing conditions, the contents of soil total N (N = 270, *H* = 9.75, *p* < 0.05), available N (N = 226, *H* = 7.93, *p* < 0.05), and soil pH (N = 144, *H* = 10.07, *p* < 0.01) in GPHPs were significantly lower than those in GDPs and BDPs; however, there were no significant differences between GDPs and BDPs in terms of these three indicators (Figure 5; Table S8).

## 4. Discussion

The field survey findings indicate that most cattle and sheep are freely grazed in natural grasslands and forests, moving unrestrictedly through the experimental and core zones of the reserve. This behavior can cause severe damage to wildlife habitats [39]. The comparison of results from the MaxEnt model under two hypotheses shows that livestock activities not only significantly reduce the total area of suitable habitats for giant pandas within the reserve (a reduction of 20.85%) but also decrease the area of highly suitable habitats (from 23.19 km^2^ to 19.91 km^2^). This leads to some areas progressively degrading to moderate or low suitability, until, in some cases, they are no longer suitable for panda habitation. This degradation directly demonstrates the destructive impact of grazing disturbance on habitat suitability, with the most significant effects observed in moderate- and low-suitability habitats. By examining the distribution of suitable habitats under H1 and H2 in Figure 2, we found that the high-suitability habitats for giant pandas are not simply reduced; some moderate and low-suitability habitats show a transformation into high-suitability habitats. Excluding the limitations of the model itself, this indicates that under the disturbance of grazing, giant pandas may choose to survive in lower-suitability habitats. The reduction in suitable habitats drives changes in the habitat selection of giant pandas.

Under natural conditions, giant pandas typically prefer habitats with lower shrub density [42,55], wide bamboo coverage, and taller, thicker bamboo [31,56]. However, our results indicate that under the influence of grazing, giant pandas tend to select habitats at higher elevations, with taller trees, more shrubs, and lower bamboo coverage. Relevant studies have shown that wild giant pandas not only choose high-altitude areas to avoid intense human disturbances in lower-altitude regions [20], but also select areas with more shrubs and lower-quality bamboo forests to avoid grazing disturbances [57,58,59,60]. Grazing has had a severe impact on the habitat and habitat selection of giant pandas. Without effective control, grazing will further reduce the area of suitable habitats for giant pandas, posing a potential threat to their survival in this region.

The diet of wild giant pandas is extremely limited, with pandas having evolved over eight million years from a carnivorous diet to specializing in bamboo consumption [12,61,62]. Therefore, the growth conditions and nutritional quality of bamboo directly affect the foraging and survival of giant pandas. Research has shown that giant pandas tend to select bamboo species with high nutritional value and good palatability [63,64,65]. However, grazing activities significantly affect the nutritional components of different bamboo species, and there are notable differences in nutrient content among different age classes. The dry matter content is an important indicator of the organic matter accumulation in bamboo and is a relatively stable type of nutrient. Under conditions without human disturbance, its content does not generally change due to panda foraging [63,66]. However, the dry matter content of *Y. ailuropodina* culms in GPHPs was significantly higher than that in GDPs, indicating that grazing reduced the dry matter content and affected the organic matter accumulation of the bamboo. This is likely due to the extensive trampling and foraging behavior of livestock, which differs from the selective foraging behavior of giant pandas, thereby impacting the nutritional components of the bamboo. Proteins and fats are essential nutrients for plant growth and development, and they are also key sources of energy for herbivores, playing an important role in their metabolism and growth. Compared to GPHPs, grazing significantly reduced the crude protein content in the perennial culms of *Y. ailuropodina* and the leaves and biennial culms of *B. faberi* in GDPs. It also decreased the ether extract content in the leaves and perennial culms of *Y. brevipaniculata* and *B. faberi*. Therefore, grazing not only reduces the crude protein and ether extract contents in bamboo but also severely affects the growth of the bamboo itself [17,24,30]. Crude fiber and crude ash are key indicators for evaluating the nutritional quality of bamboo [66] and are often used to assess its palatability. Grazing not only increases the crude fiber content in the biennial culms of *Y. ailuropodina* and the annual leaves and biennial culms of *B. faberi*, but also raises the crude ash content in the perennial culms of *Y. ailuropodina*. Due to the characteristics of the giant panda digestive system, they can only digest hemicellulose and intracellular components from food particles. Higher fiber content affects palatability and reduces the digestibility of nutrients [12]. Therefore, grazing increases the degree of fiberization in bamboo, reduces its palatability, and worsens its nutritional quality.

Animals always choose food that provides the maximum net gain to obtain an optimal diet during foraging [67]. Due to their unique physiological structure, giant pandas prefer bamboo with high crude protein and ether extract content and low crude fiber content [68,69]. Therefore, the preference for these nutritional components is an important factor in the selection of bamboo forests by giant pandas. The results of this study indicate that grazing activities have affected the five nutritional components of the primary bamboo species consumed by giant pandas in this region, with *Y. ailuropodina* being the most severely affected, followed by *B. faberi*. Grazing has reduced the amount of dry matter, crude protein, and ether extract in bamboo, while increasing the amount of crude fiber and crude ash. If it is not controlled, grazing will further reduce the nutritional quality of bamboo, which will not only affect the food acquisition of giant pandas but will also impact the growth and development of the bamboo itself.

When bamboo is subjected to grazing erosion, it releases secondary metabolites as a defense mechanism. These compounds can influence animals’ food selection and feeding frequency [70,71] and affect their growth and reproductive success by altering estrogen and prolactin levels [72]. Tannins and polyphenols affect the foraging strategies of giant pandas and are negatively correlated with their bamboo intake [31,32,73]. However, we did not find significant changes in the tannin or polyphenol contents of bamboo due to grazing. This may be due to the influence of factors such as season and light on secondary metabolites [74,75], as well as the characteristics of different bamboo species and the time span of sample collection. Under the same sampling conditions, only the flavonoid content showed significant differences under grazing conditions. Flavonoids, as important secondary metabolites, are also one of the indicators for assessing bamboo quality [76]. Bamboo with a high flavonoid content can increase progesterone and testosterone levels in giant pandas, thereby affecting their reproductive rates [33]. Additionally, the bitterness of bamboo is related to its flavonoid content [77], and the genes associated with the bitterness receptors in giant pandas have undergone positive selection, leading pandas to prefer bamboo species and parts with a stronger bitter taste [78]. Therefore, we speculate that under grazing disturbance, *B. faberi* culms might be more likely to be preferred by giant pandas compared to *Y. ailuropodina* culms. According to the 2022 bamboo data statistics from the reserve [48], although the area utilized by giant pandas for *B. faberi* and *Y. brevipaniculata* increased by a total of 7.32 km^2^ from 2018 to 2022, the proportion of *Y. ailuropodina*, the most widely distributed primary bamboo species for giant pandas, available for use by pandas decreased from 32.73% in 2018 to 12.06% in 2022, representing a reduction of 35.14 km^2^ (Table S9). Under such circumstances, giant pandas in the reserve might gradually abandon one of the largest bamboo forests.

Grazing livestock alter the bulk density [79], water content [80], and capillary water [81] of the understory soil through feeding, trampling, and excretion, which is consistent with our findings. An increase in soil bulk density makes the soil more compact, reducing water permeability and hindering plant growth [79]; this effect is associated with cumulative trampling by livestock. Additionally, livestock feeding reduces surface vegetation cover, increases surface temperature, and accelerates moisture evaporation, leading to a decrease in soil water content, which is most pronounced in BDPs. The soil capillary moisture capacity provides dissolved nutrients for plants and was found to be significantly higher in GPHPs compared to other plots subjected to grazing disturbance. Total soil C and N represent the overall nutrient reserves in the soil. In GDPs and BMPs, the significant increase in total soil N reflects that grazing enhanced soil fertility. This could be due to livestock releasing excrement into the soil, accelerating the decomposition of nitrogen-containing substances and increasing the total soil N content. In areas of continuous grazing, grazing can increase total soil N content but overgrazing can lead to a decrease in total soil N [82]. Moreover, different soil layers exhibit varied responses to grazing [83]. The primary source of total soil C is the return of plant biomass, and this carbon is influenced by multiple factors, resulting in notable spatial variation across different grassland types [84]. In our study, grazing did not result in significant differences in total soil C content. This outcome is likely closely associated with site-specific conditions, grazing intensity, soil depth, and the proliferation of C_4_ plants [85]. Compared to the relatively long-acting cycle of soil chemical properties, available P and available K can be directly absorbed and utilized by plants, making them key factors in soil fertility [86]. However, in our study, there were no significant differences in the available P and available K content, which aligns with the findings of Sun et al. [87]. Therefore, grazing has a relatively minimal impact on the levels of available P and available K in the soil. In contrast, the content of available N in GDPs and BMPs was significantly higher than that in GPHPs. This may be attributed to the reduction in forest vegetation cover under grazing conditions, which increases soil temperature and accelerates the mineralization of organic nitrogen [88]. Soil pH plays a crucial role in nutrient cycling, microbial activity, and the decomposition of organic matter [89,90]. Bamboo prefers a slightly acidic soil environment [91]. In our study, the pH values in GDPs and BMPs were significantly higher than those in GPHPs, trending towards neutral. This change may be associated with an increase in soil cations from livestock excrement, the accumulation of soluble salts in capillary moisture capacity leading to soil alkalization [92], and an increase in Hg/heavy metal content in the soil [93]. Therefore, the death of bamboo might be closely linked to the pH elevation caused by livestock excrement.

Livestock activities within the reserve are mainly concentrated from March to November each year [94], and are characterized by random foraging and diurnal behavior, resulting in grazing impacts that fluctuate over time and space [95]. Based on this, we speculate that if grazing activities in certain areas were to be reduced or ceased, giant pandas, which have a vertical distribution [96], might return to lower-altitude habitats (2000–3200 m) [59,97]. However, large-scale rubbing and trampling by livestock in bamboo forests would not only increase soil bulk density and damage the understory surface but could also reduce the soil water content and capillary moisture capacity. Additionally, livestock excrement would gradually alter the soil pH and chemical properties (such as total N content) [98], leading to a decline in the nutritional quality of the primary bamboo species consumed by giant pandas, an increase in fiber content, and reduced palatability. Livestock feeding would also hinder the photosynthesis of bamboo leaves, weakening bamboo growth [99], and induce changes in flavonoid content, leading giant pandas to prefer *B. faberi* that grows in higher-altitude areas. This change would reduce the food diversity available to giant pandas, while also making areas disturbed by grazing difficult to recover and reuse, ultimately posing a potential threat to panda survival and reproduction.

### Conservation and Management Recommendations

Grazing poses challenges to the conservation of giant pandas, affecting their survival and reproduction. Addressing the conflict between human economic development and ecological conservation requires balancing local traditions with the habitat needs of giant pandas. It is essential to designate appropriate grazing areas and strictly limit livestock access to core panda habitats, especially high-quality bamboo forests, to ensure bamboo regeneration and nutritional stability. To reduce habitat disturbance, scientific grazing management suitable for different regions should be promoted, such as reducing livestock numbers, implementing fenced grazing, and providing feed subsidies and livestock farming training to decrease community dependence on panda habitats. Additionally, it is necessary to restore bamboo forest areas damaged by grazing by replanting native bamboo species, enhancing vegetation cover, and improving soil structure and moisture retention to increase habitat sustainability. Long-term monitoring mechanisms should be established in core areas, utilizing remote sensing and drone technology to track livestock movements and habitat changes, and conducting regular assessments of soil quality, bamboo growth, and panda activity to strengthen the supervision of at-risk areas. Furthermore, ecological conservation awareness should be promoted by integrating the cultural heritage of different communities, increasing community awareness of conservation, and developing alternative economic projects such as eco-tourism to create income and reduce environmental pressure, fostering harmony between humans and nature. Lastly, strengthening cross-regional collaboration mechanisms for grazing management among different reserves can help build a unified ecological protection and management system, enhancing habitat connectivity and ensuring the overall stability of the giant panda’s living environment. Collaborative efforts should focus on incorporating the Liangshan Mountain Range into the management system of the Giant Panda National Park, laying a solid foundation for the long-term protection of giant pandas.

## 5. Conclusions

This study has revealed the profound impact of grazing on the habitat suitability and habitat quality of wild giant pandas. The MaxEnt model analysis indicated that grazing disturbance has not only significantly reduced the area of high-suitability panda habitats but also led to the progressive degradation of these areas. Some highly suitable habitats were downgraded to moderate- or low-suitability habitats, while some low-suitability areas further degraded to unsuitable habitats. The reduction in habitat suitability has forced giant pandas to migrate to higher elevations, where taller trees and more shrubs are present but the overall habitat suitability is lower, in order to avoid the disturbances caused by grazing activities. Grazing has not only affected the habitat selection of giant pandas but also utilized panda habitats with relatively taller and denser bamboo. Grazing, which is associated with livestock behaviors such as rubbing, trampling, feeding, and excretion, has led to significant changes in the physicochemical properties of the understory soil, including increased soil bulk density, decreased water content and capillary moisture capacity, and elevated levels of total N, available N, and pH. These changes have weakened the growth vitality of bamboo forests, increased the number of dead bamboo plants, affected bamboo shoot regeneration, and reduced the quality of food resources for giant pandas. Under grazing disturbance, the dry matter, crude protein, and ether extract contents of different bamboo species have significantly decreased, while the crude fiber and ash content have increased, leading to reduced nutritional quality, higher fiber content, and significantly lower palatability of bamboo. This has further impacted the quality of the giant pandas’ primary food source. Therefore, effective management of grazing activities is crucial for maintaining the ecological suitability of giant panda habitats and the sustainability of their food resources.

## Figures and Tables

**Table 1:** Comparison of flavonoid (g/kg) content in bamboo leaves and culms of three bamboo species between giant panda habitat plot (GPHP) and grazing disturbance plot (GDP).

The Types of Bamboo	Plant Parts	Grazing Disturbance Plot (GDP)	Giant Panda Habitat Plot (GPHP)	N	*t*	*p*	Cohen *d*
		Mean ± SD	Mean ± SD				
*Y. ailuropodina*	Culm	3.15 ± 0.89	4.50 ± 1.45	18	−2.37	0.031 *	1.206
	leaf	15.97 ± 7.73	16.06 ± 8.18	26	−0.03	0.978	-
*Y. brevipaniculata*	Culm	4.03 ± 1.33	4.90 ± 1.57	20	−1.31	0.205	-
	leaf	24.64 ± 6.09	24.46 ± 7.06	20	0.06	0.952	-
*B. faberi*	Culm	4.46 ± 1.46	3.03 ± 0.77	18	2.59	0.020 *	1.228
	leaf	16.95 ± 2.27	17.23 ± 10.98	18	−0.09	0.933	-

Note: * indicates a significant difference, - indicates a null value.

## Data Availability

The species occurrence data are owned by China West Normal University and the National Forestry and Grassland Administration. Access to these data requires contacting the authors or the corresponding author. Other data are available within the main text and Supplementary Materials.

## Supplementary Materials

The following supporting information can be downloaded at https://www.mdpi.com/article/10.3390/ani15020202/s1, Figure S1: Nutritional quality differences in different parts of Y. ailuropodina of various ages in response to grazing impact; Figure S2: Nutritional quality differences in different parts of B. faberi of various ages in response to grazing impact; Figure S3: Nutritional quality differences in different parts of Y. brevipaniculata of various ages and parts in response to grazing impact; Figure S4: The difference in flavonoid content between two types of samples; Table S1: Environment factors for the MaxEnt model; Table S2: Nutritional and secondary metabolite determination indexes of bamboo and their methods; Table S3: Determination index and method of soil physical and chemical properties; Table S4: Comparison of differences in variables between the GPHP and GDP; Table S5: Comparison analysis of tannin content (g/kg) of three types of bamboo in two types of plots; Table S6: Comparison analysis of polyphenols (g/kg) in three types of bamboo in two sample plots; Table S7: Comparison of soil physical properties under different forest types; Table S8: Comparison of soil chemical properties under different forest understory types; Table S9: Statistical information on bamboo in Megu Dafengding National Nature Reserve.
