# Supplementary material for: Grazing-Induced Habitat Degradation: Challenges to Giant Panda Survival Resulting from Declining Bamboo and Soil Quality
Source: Animals (Basel). 2025 Jan 14;15(2):202. doi: 10.3390/ani15020202 (PMC11758315; doi:10.3390/ani15020202)
Supplement: Supplementary file 1 [file animals-15-00202-s001.zip › animals-3360090-supplementary.pdf]

Supplementary Material

Table S1. Environment factors for the MaxEnt model.

Environmental factors		Source	Type of variable
Climate	annual average precipitation	https://www.openstreetmap.org	Continuous
Topography	Digital Elevation Model (DEM)	https://www.gscloud.cn	Continuous
	Aspect		Continuous
	Slope		Continuous
Vegetation	Normalized Difference Vegetation Index (NDVI)	https://www.earthdata.nasa.gov	Continuous
Land cover	China Land Cover Dynamics（CLCD）	https://zenodo.org	Categorical
Human activities	Grazed	Euclidean distance from grid to grazing disturbance points is calculated by ArcGIS	Continuous

Table S2. Nutritional and secondary metabolite determination indexes of bamboo and their methods.

Measurement indicators	Measurement method.	Instrument
Crude protein	Kjeldahl method	Haineng SOX500 Fat Extractor, Shandong Haineng Instrument
Crude fiber	Van Slyke method	ANKOM A200i Semi-Automatic Fiber Analyzer, ANKOM Company in the United States
Ether extract	Soxhlet extraction method	Haineng SOX500 Fat Extractor, Shandong Haineng Instrument
Crude ash	Dry ashing	Thermo Scientific Lindberg / Blue M Moldatherm 1 100°C Box-type Muffle Furnace
Dry matter	Drying method	Oven
Tannin	UV spectrophotometry method	UV Spectrophotometer
Flavonoids.	UV spectrophotometry method	UV Spectrophotometry
Polyphenols	Ferrous tartrate colorimetric method	Spectrophotometer

Table S3. Determination index and method of soil physical and chemical properties.

Measurement indicators	Measurement method.	Instrument
Available N	Alkali decomposition diffusion method	Diffusion dish
Available K	Ammonium acetate leaching-flame photometric method	Flame photometer
PH	Potentiometric method	pH meter
Available P	0.5mol/L sodium bicarbonate leaching-molybdenum antimony ratio colorimetric method	Spectrophotometer
Total N	Carbon nitrogen analyzer determination method	Carbon and nitrogen analyzer
Total C	Carbon nitrogen analyzer determination method	Carbon and nitrogen analyzer
Bulk density	Ring knife method	Ring knife specification 50.46x50 mm model 100 cm3
Water content	Aluminum box drying method	Aluminum box specification 70x38 mm
Water holding capacity	Ring knife water absorption method	Ring knife specification 50.46x50 mm model 100 cm3
Capillary porosity	Ring knife water absorption method	Ring knife specification 50.46x50 mm model 100 cm3

Note: Soil Total C 100 mesh, Soil Total N 100 mesh, Soil Available N 18 mesh, Soil Available P 18 mesh, Soil Available K 18 mesh, pH value 18 mesh.

Table S4. Comparison of differences in variables between the GPHP and GDP.

Variable	Grazing Disturbance Plot (GDP)	Giant Panda Habitat Plot (GPHP)	N	*t*/*Z*	*p*	Cliff's delta/ Cohen
	Mean ± SD/Median (Q1,Q3)	Mean ± SD/Median (Q1,Q3)				|δ|/ *d*
Elevation(m)	2719.00(2434.75, 2988.25)	2848.50(2649.50, 3057.00)	1338	-5.91	0.000*	0.198
Slope(°)	2.00（2.00, 3.00）	2.00（2.00, 3.00）	1327	-1.72	0.086	
Slope position	5.00(2.00, 5.00)	5.00(3.00, 5.00)	1238	-0.97	0.332	
Aspect	5.00(2.00, 6.00)	5.00(2.00, 6.00)	1328	-0.42	0.672	
Number of trees	23.5 ± 17.32	29.63 ± 15.31	56	-1.70	0.089	
Tree height (m)	15.00(11.00, 18.00)	16.00(12.00, 20.00)	1228	-5.21	0.000*	0.182
Tree diameter at breast height (cm)	20.00(15.00, 28.00)	20.00(16.00, 30.00)	1223	-2.90	0.004*	0.102
Canopy closure of trees (%)	2.00(2.00, 3.00)	2.00(2.00, 3.00)	1303	-1.54	0.123	
Number of shrubs	32.16 ± 14.36	41.81 ± 13.50	57	-2.61	0.012*	0.690
Shrub height (m)	4.00(3.00, 5.00)	4.00(3.00, 5.00)	1267	0.29	0.770	
Shrub cover (%)	3.00(3.00, 3.00)	3.00(3.00, 3.00)	1312	-1.72	0.086	
Bamboo height (m)	3.00(2.00, 4.00)	3.00(2.00, 4.00)	1312	2.02	0.043*	0.065
Bamboo cover (%)	2.00(2.00, 3.00)	2.00(2.00, 3.00)	1303	3.72	0.000*	0.116
Bamboo growth status	2.00(2.00, 2.00)	2.00(2.00, 2.00)	1309	-4.80	0.000*	0.048
Number of dead bamboo plants	2.50(0.00, 4.50)	0.00(0.00, 2.00)	54	2.71	0.007*	0.412
Number of shoots	0.00(0.00, 0.00)	4.00(0.50, 7.00)	55	-4.63	0.000*	0.661

Note: *Significant difference.

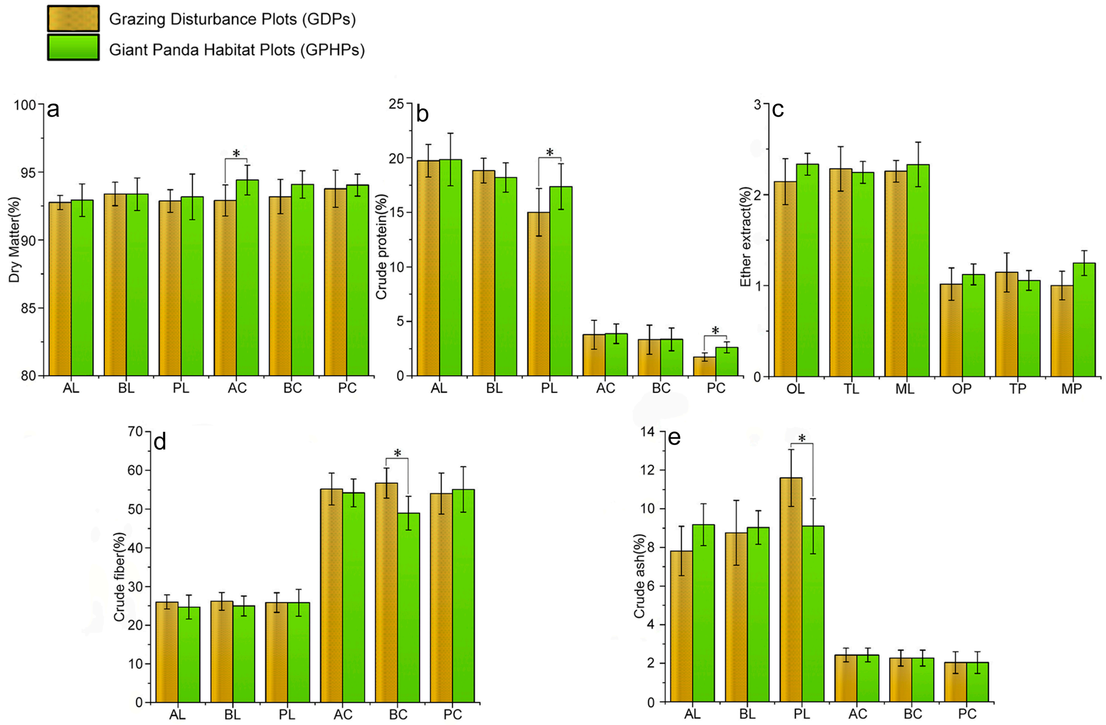

Figure S1. Nutritional quality differences in different parts of *Y. ailuropodina* of various ages in response to grazing impact (Nutritional quality data were tested for normality, and most data showed normal distribution. Therefore, Mean ± Standard Deviation (SD) was used to construct bar charts with error bars to uniformly illustrate changes in nutritional quality. Abbreviations in the figure: AL: annual leaves, BL: biennial leaves, PL: perennial leaves, AC: annual culms, BC: biennial culms, PC: perennial culms; a. dry matter, b. crude protein, c. ether extract, d. crude fiber, e. crude ash. Significance levels: * $p < 0.05$, indicating significant differences.).

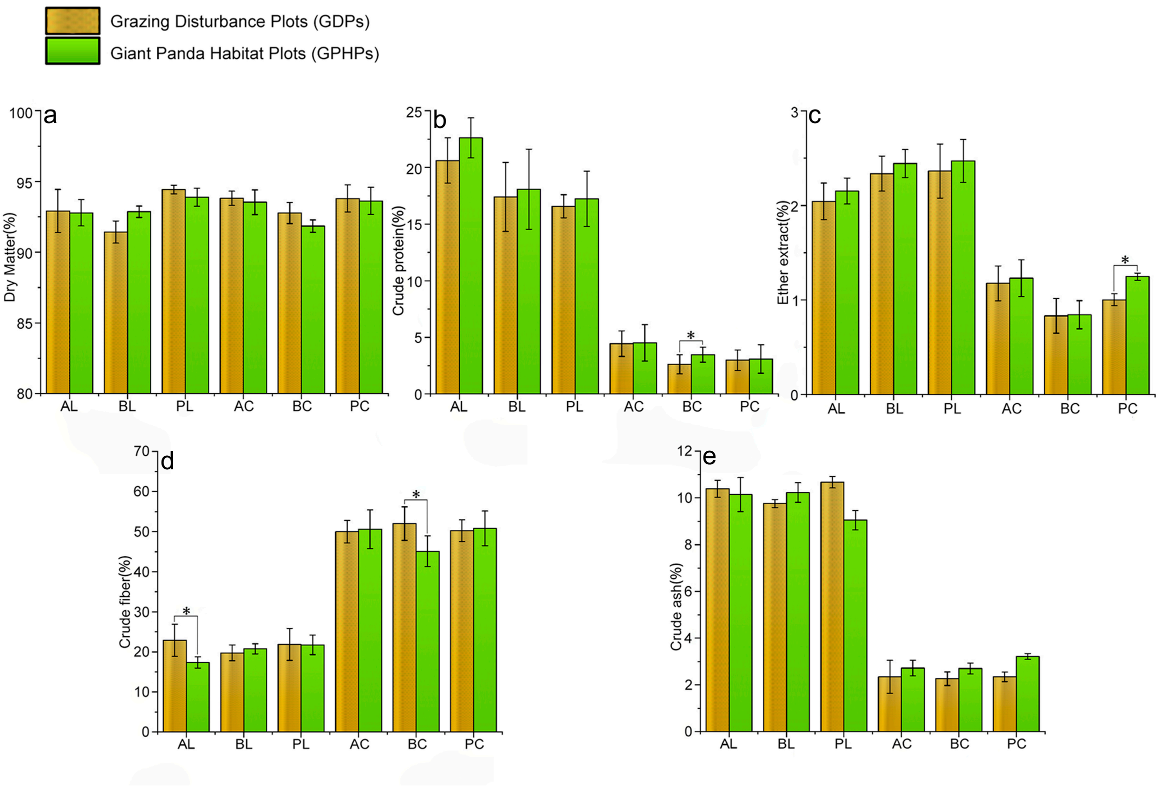

Figure S2. Nutritional quality differences in different parts of *B. faberi* of various ages in response to grazing impact (Nutritional quality data were tested for normality, and most data showed normal distribution. Therefore, Mean ± Standard Deviation (SD) was used to construct bar charts with error bars to uniformly illustrate changes in nutritional quality. Abbreviations in the figure: AL: annual leaves, BL: biennial leaves, PL: perennial leaves, AC: annual culms, BC: biennial culms, PC: perennial culms; a. dry matter, b. crude protein, c. ether extract, d. crude fiber, e. crude ash. Significance levels: * $p < 0.05$, indicating significant differences.).

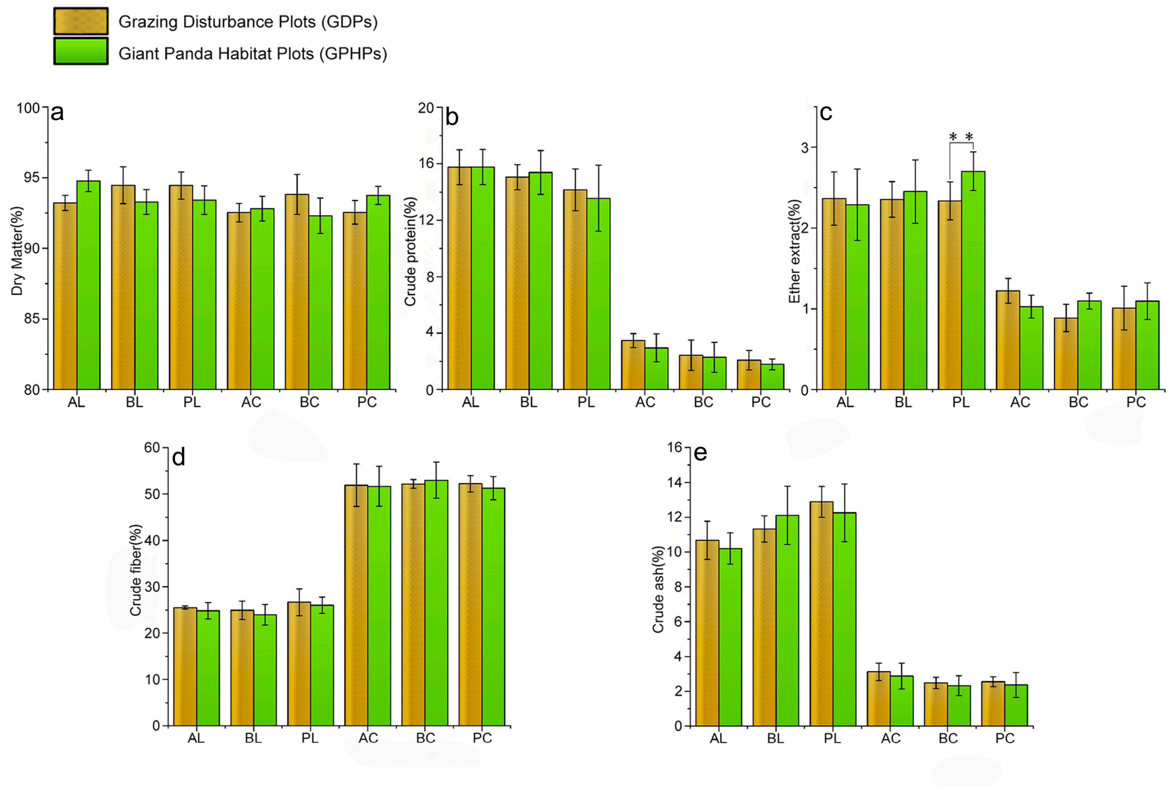

Figure S3. Nutritional quality differences in different parts of *Y. brevipaniculata* of various ages and parts in response to grazing impact (Note: Nutritional quality data were tested for normality, and most data showed normal distribution. Therefore, Mean ± Standard Deviation (SD) was used to construct bar charts with error bars to uniformly illustrate changes in nutritional quality. Abbreviations in the figure: AL: annual leaves, BL: biennial leaves, PL: perennial leaves, AC: annual culms, BC: biennial culms, PC: perennial culms; a. dry matter, b. crude protein, c. ether extract, d. crude fiber, e. crude ash. Significance levels: * $p < 0.05$, indicating significant differences.).

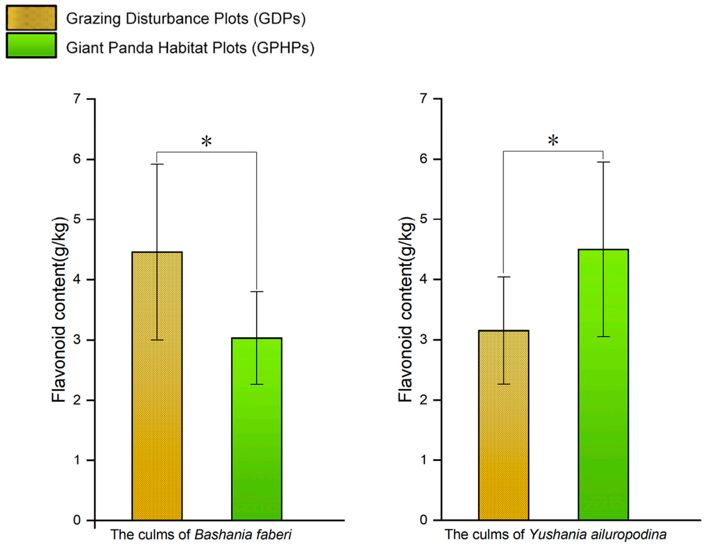

Figure S4. The difference in flavonoid content between two types of samples (Due to the normality of the data, bar charts with Mean ± SD are used to present the results. Significance levels: * $p < 0.05$, indicating significant differences.).

Table S5. Comparison analysis of tannin content (g/kg) of three types of bamboo in two types of plots.

The types of bamboo	Plant parts	Grazing Disturbance Plot（GDP）	Giant Panda Habitat Plot(GPHP)	N	*t/Z*	*p*
		Mean ± SD/Median (Q1,Q3)	Mean ± SD/Median (Q1,Q3)			
Y. ailuropodina	culm	4.30 ± 1.49	3.37 ± 1.25	18	1.43	0.173
	leaf	9.62 ± 5.30	10.54 ± 4.74	27	-0.47	0.646
Y. brevipaniculata	culm	4.84 ± 1.47	3.58 ± 1.92	21	1.71	0.103
	leaf	15.03 ± 5.11	14.51 ± 2.80	21	0.30	0.768
B. faberi	culm	4.04(2.94, 4.14)	4.20(3.95, 5.36)	18	-1.63	0.102
	leaf	9.20 ± 4.52	9.81 ± 1.63	18	-0.42	0.683

Table S6. Comparison analysis of polyphenols (g/kg) in three types of bamboo in two sample plots.

The types of bamboo	Plant parts	Grazing Disturbance Plot（GDP）	Giant Panda Habitat Plot(GPHP)	N	*t/Z*	*p*
		Mean ± SD/Median (Q1,Q3)	Mean ± SD/Median (Q1,Q3)			
Y. ailuropodina	culm	10.32 ± 1.82	11.99 ± 1.85	18	-1.93	0.071
	leaf	20.20 ± 4.11	19.95 ± 5.05	26	0.14	0.892
Y. brevipaniculata	culm	11.36 ± 1.86	12.74 ± 2.05	20	-1.56	0.137
	leaf	27.82(23.46, 29.85)	23.63(22.06, 30.84)	20	0.08	0.939
B. faberi	culm	11.96 ± 1.80	11.15 ± 1.39	18	1.07	0.300
	leaf	20.39 ± 2.36	20.92 ± 7.07	18	-0.24	0.817

Table S7. Comparison of soil physical properties under different forest types.

Variables	Giant Panda Habitat Plot（GPHP）	Grazing Disturbance Plot（GDP）	Bamboo Death Plot (BDP)	*H*	N	*p*	Adjusted *p* (Bonferroni)
Soil water content（%）	55.48(39.77, 66.27)	47.18(38.86, 65.01)	43.58(35.75, 53.84)	6.59	259	0.037	0.031
Soil capillary porosity（%）	49.30(43.70, 52.62)	49.47(45.89, 53.56)	50.10(46.54, 53.59)	0.68	191	0.712	—
Soil capillary moisture capacity（%）	70.70(68.33, 77.65)	62.26(53.81, 68.90)	64.96(58.52, 73.68)	10.53	126	0.005	0.004
Soil bulk density（g/cm^3）	1.57(1.37, 1.75)	1.62(1.51, 1.80)	1.64(1.54, 1.82)	6.52	227	0.038	0.038

Table S8. Comparison of soil chemical properties under different forest understory types.

Variables	Giant Panda Habitat Plot（GPHP）	Grazing Disturbance Plot（GDP）	Bamboo Death Plot (BDP)	*H*	N	*p*	Adjusted *p* (Bonferroni)
Soil Total N（%）	0.68(0.47, 1.01)	0.91(0.58, 1.25)	0.92(0.61, 1.24)	9.75	270	0.008	0.032/0.031
Soil Total C（%）	8.13(5.59, 10.82)	7.94(5.81, 11.50)	7.97(5.90, 11.25)	0.97	272	0.616	—
Soil Available K（mg/Kg）	289.00 (158.00, 388.00)	259.00 (175.00, 359.00)	259.00 (170.25, 351.00)	0.27	153	0.873	—
Soil Available N（mg/Kg）	814.38 (586.53, 996.66)	618.87 (313.85, 962.85)	814.38 (649.74, 996.66)	7.93	226	0.190	0.033
Soil Available P（mg/Kg）	2.10(1.13, 22.37)	3.05(1.47, 9.43)	2.85(1.21, 10.38)	0.17	154	0.918	—
Soil PH	4.34(4.03, 4.82)	4.88(4.21, 5.74)	5.00(4.09, 5.71)	10.07	144	0.007	0.008

Table S9. Statistical information on bamboo in Megu Dafengding National Nature Reserve.

Bamboo species	Altitude（m）	Area（km^2）	The Giant panda utilization of area（km^2）	
			Before 2018	2018-2022 years
Yushania ailuropodina	1356-2821	169.88	55.63	20.49
Bashania faberi	2845-3771	133.83	25.04	28.71
Yushania brevipaniculata	2454-3297	111.14	41.52	45.17
Yushania dafengdingensis	2816-3098	20.03	20.03	—
Yushania mabianensis	2562-2816	3.35	3.35	—
Chimonobambusa szechuanensis	2070-2170	2.63	—	—

Note: After 2018, giant pandas in Meigu Dafengding have been moving westward, with little change in latitude, resulting in a decrease of 51.2 km^2 in bamboo utilization area.

Table reference: Gu, Y.; Chen, M.; Zeng, J.; Ma, X. Study on the Relationship Between Bamboo Resources and Giant Panda Activity Areas in Meigu Dafengding National Nature Reserve. Sichuan Forestry Sci. Technol. 2022, 43, 24–30.
